# Potential of digital technologies in counteracting long-standing deficits in hemodialysis machine training

**DOI:** 10.1038/s41598-025-89435-w

**Published:** 2025-02-14

**Authors:** Maximilian Rettinger, Julia Steinhaus, Annika Hackenberg, Lisa Lehr, Niklas Müller, Matthias Schöffel, Sonja Pandit, Julia Mayer, Christopher Holzmann-Littig, Gerhard Rigoll, Christoph Schmaderer

**Affiliations:** 1 Chair of Human-Machine Communication, Technical University of Munich, Munich, Germany; 2 University Hospital rechts der Isar, Technical University of Munich, Munich, Germany

**Keywords:** Health care, Risk factors

## Abstract

Before medical professionals are permitted to use a medical device, they first must be instructed in its use. However, it is well known that this method is hazardous for both the staff and the patients due to its inadequate quality. In order to address this problem, we investigated the potential of digital technologies for enhancing medical device training. For this, we designed and implemented several diverse training methods: (1) conventional training by a medical instructor, (2) video-based training, (3) mobile application training on a tablet, (4) virtual reality training, and (5) augmented reality training. Since each method provides identical training content to the user, we compared the resulting learning outcomes between the methods. The findings indicate that virtual and augmented reality training is superior to conventional training. These digital technologies offer the opportunity to reduce the burden on healthcare professionals and increase patient safety.

## Introduction

**Fig. 1:**
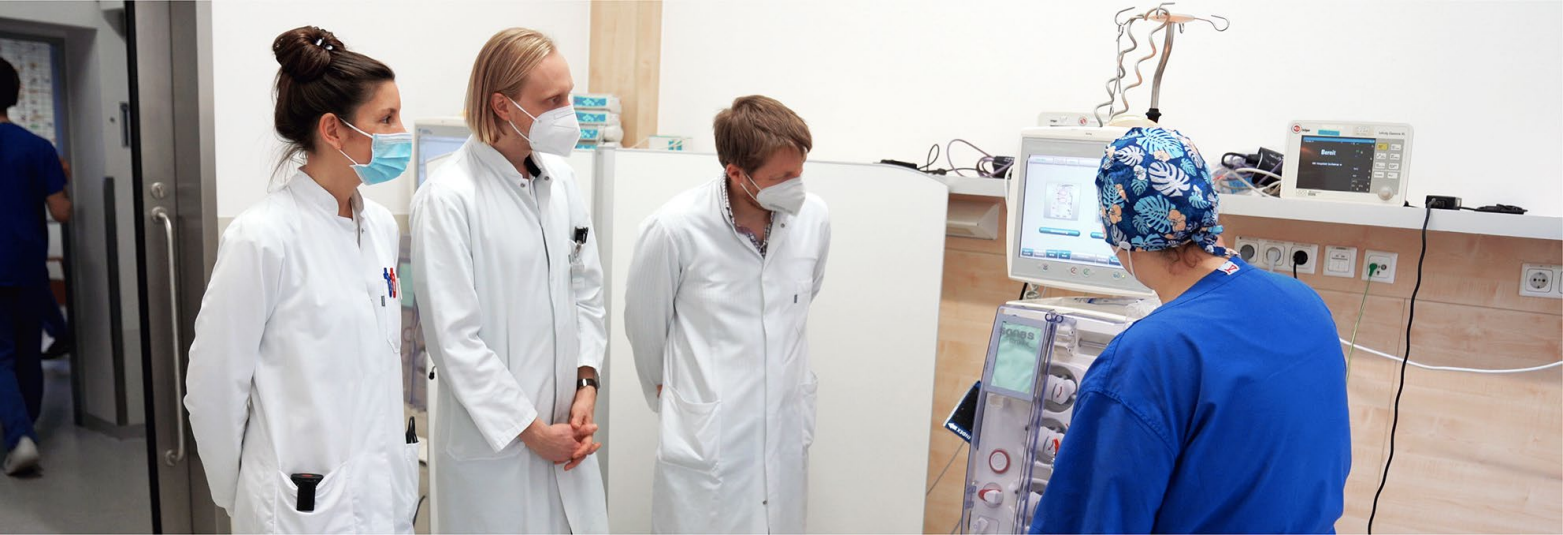
Excerpt of the conventional training for priming a dialysis machine. The depicted number of participants is considerably lower than usual.

Medical facilities have a variety of medical devices that require staff training to ensure their proper use. Typically, this legally required training is conducted on-site by an expert from the manufacturing company. However, studies and expert reports from different countries have revealed that this type of training is insufficient and compromises patient safety^1–9^. This is not a new problem, as such findings have been known to the public for decades, and with the increasing complexity of medical devices^4^, no improvement is expected^10,11^. There are many underlying reasons, such as organizational and social barriers^5,12^, but also short and infrequent training sessions caused by financial and time constraints^13^, resulting in large training groups and complicated scheduling. Since the devices are usually immobile (*e.g*., due to their size or connectivity) and large rooms are unavailable^14,15^, the training typically occurs in small spaces. As a result, situations may arise in which the participant’s field of vision is blocked by other medical devices or other people in the room. Staff shortages or time constraints force trainees to choose between patient care or the legally required equipment training^16^. Ambient noises, such as device sounds or other people, negatively affect trainees’ performance^17^. In addition, the opportunity to apply or train newly learned abilities to the medical device is often limited, e.g., since the staff has no time for it afterward or all devices are already in use with patients. Extended reality approaches such as virtual reality (VR), augmented reality (AR), or even a mobile application (MA) have the potential to avoid these problems but also offer advantages such as reusability, multilingualism, or standardization, which can reduce errors^2^. Especially during emergency situations like pandemics, digital approaches can offer life-saving benefits^18^. Experts have recommended integrating better training methods for years^19,20^, but so far, they hardly exist^3,13^ (Fig. 1).

Extended reality approaches for improving medical education have been a popular research topic for years^21,22^. We base our work on a contribution that examined the difference between traditional instructor-based training, video training, and single-user VR training^23^. Study results indicated that training in the virtual environment performs better than the other methods. Furthermore, additional research comparing single-user and multi-user VR training with the traditional approach has demonstrated that virtual training-whether conducted individually without an instructor or with one and other participants-consistently outperforms the traditional training method^24^. There are also more reports on VR training in the medical field, presenting the technology, but few empirical results prove the potential of VR training^25,26^. The work by Lee et al.^27^ examined the use of peritoneal dialysis. They compared printed educational materials with a 3-dimensional application running on a laptop computer. The results showed that the performance was significantly better in the digital group, which all participants preferred. Considering that a tablet or smartphone application is more flexible than a computer, we examine a mobile tablet application in this investigation. Heo et al.^28^ studied two methods for setting up a medical ventilator. The conventional group completed the training with a printed manual and received telephone support, while the other group used AR (with a Microsoft HoloLens 2). Participants in the AR group were more confident and required less assistance than those in the manual group.

Digital solutions for enhancing the training of complex tasks such as manual assembly or machine maintenance offer the opportunity to reduce task duration or rather save personnel resources^29,30^. As the training procedures are similar to the priming of a medical device, the current state of development and evaluation of industrial training systems is also relevant to this contribution. Winther et al.^31^ compared the training methods: (1) Conventional pairwise, (2) Video, and (3) VR training for a maintenance task on a dosing pump. There were 19 identical training steps in each method. The results of a study with 36 participants indicated that VR training takes significantly more time and has a higher error rate. According to the authors, the results suggest that AR training with animations and step-by-step instructions may be superior, e.g., via AR projection^32^ or an AR HMD^33^. Werrlich et al.^33^ investigated this using an AR HMD (Microsoft HoloLens) and compared it with paper instructions for training an assembly process. The results of their experiment ($n=30$) revealed that the conventional paper method is significantly faster. However, the results also indicated significantly fewer errors in the AR training. In a similar study ($n=20$), Moghaddam et al.^34^ compared paper and AR training (using a Microsoft HoloLens 2) for assembly tasks. The results showed that the training success with AR was significantly better than with the traditional method. Another paper by Liu et al.^35^ examined video, tablet, and AR training (using Microsoft HoloLens 2) for training maintenance tasks. The findings of a study with 60 participants indicated that AR training performed better with increasing complexity compared to the other methods. Funk et al.^36^ investigated the following four systems for providing assembly instructions at the workplace: (1) Paper instructions printed on several A4 paper sheets, (2) HMD instructions presented to the user via a see-through HMD (Epson Moverio BT-200). These instructions were the same images as in the Paper Condition, and the next step occurred when the current one was solved correctly (using the Wizard of Oz technique^37^). (3) Tablet instructions displayed the same images as the Paper method on a Tablet, and the content was controlled externally, as in the HMD method. (4) In-situ instructions were provided with a projector at the assembly position. A study with 16 participants showed that locating a part was significantly faster with the conditions In-situ and Paper. The perceived cognitive load was significantly lower for the In-situ instructions than for the HMD. Regarding the errors, participants had significantly more mistakes with the HMD than with the tablet and the In-situ method. A similar work by Büttner et al.^38^ compared the methods: (1) Paper, (2) Personal, and (3) In-situ. The results showed that Personal training was the most efficient due to its speed. However, a knowledge measurement indicated no differences between the groups. Zheng et al.^39^ showed that pictorial instructions placed in the center of a see-through HMD (using Epson Moverio BT-200) were better than in a peripheral position. Participants preferred instructions via the HMD due to increased comfort and reduced distraction. Blattgerste et al.^40^ compared paper instructions with three technological devices for presenting instructions. These were a smartphone, an Epson Moverio BT-200 (the same one used by Funk et al.^36^ and Büttner et al.^38^), and a Microsoft HoloLens (same as Werrlich et al.^33^, Moghaddam et al.^34^, and Liu et al.^35^). The findings indicated that the error rate was significantly lower with the HoloLens, while the training duration between the methods was comparable. Apart from that, the cognitive load for the conventional paper training was the lowest. We also investigate AR as a training method since it is similar to the In-situ method and thus offers the possibility of displaying relevant information at the correct position. This also means that the user’s field of view is less restricted since the visual information is directly referred to the real environment and not to a superimposed image as in the HMD method. Since the In-situ method is very complex in terms of construction and flexibility, it is not suitable as a future training method in medical institutions and is therefore not considered.

Overall, digital technologies offer many opportunities and are widely explored and used in training areas such as manufacturing^41,42^ or safety training^43,44^. The need to improve medical device training is obvious, but it is unclear whether available technologies are suitable and can actually improve the training outcome. Most existing research contributions focus on comparing a conventional training method with one or at most two alternative approaches, leaving the differences between other technologies largely unexplored. To close this gap, we are the first to implement five comparable training methods with identical training content and examine their suitability in a comprehensive study ($n=140$) in order to answer the following research questions:

- RQ 1: Can alternative training methods improve medical device training?
- RQ 2: Do alternative medical device training methods differ in terms of training success?

Our use case focuses on the priming procedure of a hemodialysis machine, for which we design, implement, and evaluate the following methods: (1) *Group* training (conventional training by an instructor), (2) *Video* training (video recording of the training content), (3) *MA* training (mobile application for interactive priming of a digital device), (4) *VR* training (interactive priming for a machine in a virtual environment), and (5) *AR* training (priming for a real machine augmented with virtual content). Studies that have compared different training methods already exist^45^. However, the specific feature of this contribution is that we investigate possibilities for solving a long-standing problem by comparing the *most* feasible training methods (*Video*, *MA*, *VR*, and *AR*) for device training in medical facilities. To the best of our knowledge, there has been no direct comparison of different training methods in such an extensive sample. The resulting findings help to identify the potential of alternative approaches for improving the future effectiveness of medical device training and ultimately increasing patient safety.

## Results

### Preliminary study

We conducted a preliminary study ($n=15$) to evaluate the experience (*e.g*., functionality, usability) of the *MA*, *VR*, and *AR* conditions and to optimize them accordingly. Results showed that users of the *MA* training had problems activating virtual switches, so the sensitivity was increased. In the *VR* training, participants complained of light nausea and vertigo, which are cybersickness symptoms^46^. We countered this by reducing the user’s bending motion by placing objects on the virtual table that were previously underneath it.

During the *AR* training, all five participants experienced problems pressing the virtual button to initiate the next training step. This is due to inaccuracies in hand tracking^47^ and incorrect distance judgments^48^. As a solution, we replaced the button with a speech recognition control that triggers the subsequent task using the voice command ’next’.

### Procedure

First, participants’ demographic data were collected after the study procedure was explained and the consent form was signed. Next, they received information and instructions about the respective training method and its relevant hardware. Participants of the *Video*, *VR*, or *AR* method also underwent a tutorial to familiarize themselves with the system. Subsequently, they completed the dialysis training, followed by another questionnaire. Finally, participants were informed that they would receive an online test in one week, which they would have to solve honestly and without help (*e.g*., from colleagues, online search engines, and books). One week later, the participants received the online test by e-mail, and after completing it, they were remunerated with the equivalent of $16.5 for their participation. The study took approximately 45 minutes, and each training method was performed without any time limit.

### Statistics

The experiment’s results are statistically compared using one-way analysis of variance (ANOVA) and subsequent post-hoc Tukey’s HSD tests. A Welch correction was applied when Levene’s test indicated a violation of homogeneity. A significance level of $\alpha = 0.05$ was used for all tests. The significance between conditions is marked in the Figures of this section; the p-values are presented as ($p <.05$)*, ($p <.01$)**, and ($p <.001$)***.

### Participants

**Table 1:** Participants’ distribution to the respective training groups.

Method	n	Age		Gender		Medical profession		
		M	SD	Male	Female	Doctor	Nurse	Student/PhD
Group	28	28.57	7.41	8	20	7	3	18
Video	28	29.00	9.43	9	19	5	10	13
MA	28	28.46	8.24	7	21	5	9	14
VR	28	33.89	11.02	11	17	8	11	9
AR	28	28.86	10.15	11	17	2	5	21
$\Sigma$	140	29.76	9.44	46	94	27	38	75

We recruited 140 participants (94 female, 46 male) via mailing lists, social networks, direct outreach, and flyers distributed to different clinics. The study was conducted on the premises of a university hospital, ensuring that participants had sufficient space and were undisturbed. In order to obtain a comparable distribution across the five training methods, we divided the participants based on their demographic data, as shown in Table 1. Their ages ranged between 18 and 59 years ($M = 29.76$, $SD = 9.44$). Regarding technical experience, each of the 28 participants in the *MA* group had previously interacted with software applications on smartphones/tablets. Six participants in the *VR* group already had previous experience with this immersive technology. In the *AR* group, two participants had experience with the augmented physical real world. All participants had an active medical background since the use case was training for a medical product. This means that only individuals from the professional groups of physicians, nurses, and medical students/doctoral fellows participated in this study. In addition, individuals who had already undergone dialysis training were excluded.

### Training duration

First, we analyzed the average completion time in minutes participants needed for the training. The Welch’s ANOVA indicated a significant difference between the training durations in minutes $$F(4, 60.246) = 167.835,$$ $$p <.001$$, $$\eta ^{2} = 0.802$$. Pairwise comparisons showed that the *MA* training ($$M = 6.77$$, $$SD = 1.55$$) took the least completion time, followed by *Video* ($$M = 7.02$$, $$SD = 2.64$$), *Group* ($$M = 8.39$$, $$SD = 0.58$$, with *MA* $$p =.027$$), *VR* ($$M = 10.10$$, $$SD = 2.68$$, all $$p<=.016$$), and *AR* $$(M = 17.62$$, $$SD = 1.89$$, all $$p <.001)$$ training.

### Specific questions

**Fig. 2:**
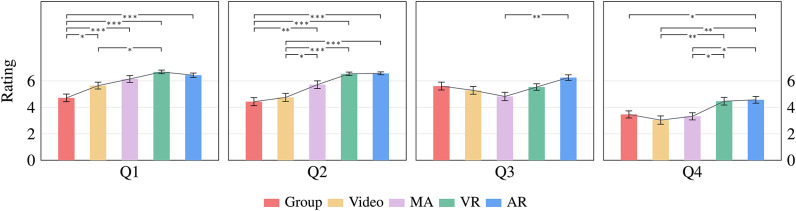
Mean ratings of the first four specific questions using a 7-point Likert scale [1 = strongly disagree, 7 = strongly agree]. The Error bars represent the standard error.

**Fig. 3:**
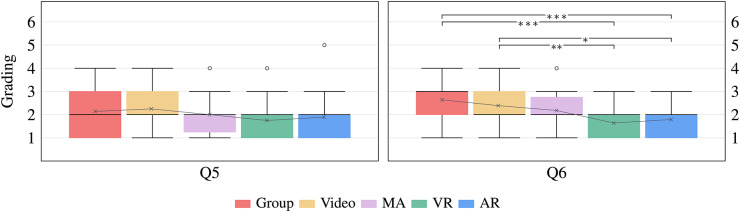
Illustration of the last two specific questions using a 6-point Likert scale [1 = very good, 6 = not sufficient]. The horizontal line in each box indicates the median, the lower and upper limits of the box represent the $25^\textrm{th}$ and $75^\textrm{th}$ percentiles, single outliers are visualized as gray circles, and the connected crosses are the mean values.

Figure 2 presents the descriptive results of questions (Q1 to Q4), where higher values indicate a positive evaluation. Conversely, lower values indicate a positive evaluation for the results illustrated in Fig. 3, as these questions (Q5 and Q6) were rated with grades.

Q1: “*I think this training was clearly structured.*” For the ratings of this question, Welch’s ANOVA indicated significant differences between the groups $F(4, 65.961) = 10.842$, $p <.001$, $\eta ^{2} = 0.254$. Accordingly, the comparison between the conditions indicated that the structure of all groups was rated significantly (all $p <=.038$) better compared to the *Group* training ($M = 4.71$, $SD = 1.54$). In addition, the *VR* training ($M = 6.68$, $SD = 0.72$) received the highest scores and was also rated significantly ($p =.015$) better than the *Video* training ($M = 5.64$, $SD = 1.37$).

Q2: “*I think the training topic was interestingly prepared.*” Welch’s ANOVA indicated a significant main effect for the preparation of the training $$F(4, 64.429) = 18.652$$, $$p <.001$$, $$\eta ^{2} = 0.328$$. The *AR* $$(M = 6.57,$$ $$SD = 0.57),$$ *VR* ($$M = 6.54$$, $$SD = 0.69$$), and *MA* training ($$M = 5.71$$, $$SD = 1.54$$) were significantly (all $$p <=.047$$) more appealing than the *Video* ($$M = 4.75$$, $$SD = 1.60$$) and *Group* training ($$M = 4.43$$, $$SD = 1.62$$).

Q3: “*I think the training was close to reality.*” A one-way ANOVA showed a significant difference between the training methods $F(4, 135) = 3.565$, $p = .008$, $\eta ^{2} = 0.096$. The *AR* condition ($M = 6.25$, $SD = 1.11$) had the highest ratings and significantly ($p =.003$) differed from the *MA* condition ($M = 4.82$, $SD = 1.68$) in the post-hoc test.

Q4: “*I feel able to prime the dialysis machine without any problems.*” For this question, we found that the five conditions significantly impacted the subjective ability of priming the machine $F(4, 135) = 6.213$, $p = .001,$ $\eta ^{2} = 0.155$. The *AR* training ($M = 4.57$, $SD = 1.35$) received the best ratings and differed significantly (all $p <=.047$) compared to the *Group* ($M = 3.46$, $SD = 1.43$), *Video* ($M = 3.04$, $SD = 1.67$) and *MA* training ($M = 3.32$, $SD = 1.44$). In addition, the *VR* training ($M = 4.46$, $SD = 1.53$) was also significantly (all $p <=.037$) better than the *Video* and *MA* training.

Q5: “*Which grade would you give the quality of the content?*” In terms of content quality, no significant differences were observed between the methods $F(4, 135) = 1.532$, $p = .196$, $\eta ^{2} = 0.043$. On average, the *VR* ($M = 1.75$, $SD = 0.80$) and *AR* ($M = 1.89$, $SD = 0.83$) conditions received the best rating, followed by *MA* ($M = 2.00$, $SD = 0.82$), *Group* ($M = 2.14$, $SD = 0.93$), and *Video* ($M = 2.25$, $SD = 0.84$).

Q6: “*Which grade would you give the training overall?*” The one-way ANOVA indicated a significant difference in the overall assessment of the training methods $F(4, 135) = 8.773$, $p <.001$, $\eta ^{2} = 0.206$. Overall, the *VR* ($M = 1.64$, $SD = 0.56$) and *AR* training ($M = 1.79$, $SD = 0.63$) received the best ratings and indicated a significant difference compared to the *Group* ($M = 2.64$, $SD = 0.91$, all $p <.001$) and *Video* training ($M = 2.39$, $SD = 0.79$, all $p <=.022$).

### Workload

**Fig. 4:**
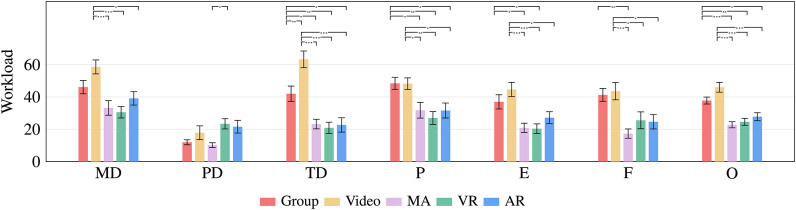
Mean scores of the NASA-RTLX assessments ranging from 0 [very low] to 100 [very high]. The scales are (MD) Mental demand, (PD) Physical demand, (TD) Temporal demand, (P) Performance, (E) Effort, (F) Frustration, and (O) Overall. Error bars depict the standard error.

The ratings of the six RTLX subscales and the overall score, depicted in Fig. 4, were statistically compared between the conditions as follows:

MD: “*Mental demand - How mentally demanding was the task?*” For Mental demand, ANOVA showed significant differences $F(4, 135) = 7.377$, $p <.001$, $\eta ^{2} = 0.179$. The *MA* ($M = 33.21$, $SD = 23.97$), *VR* ($M = 30.54$, $SD = 18.73$), and *AR* ($M = 39.11$, $SD = 21.90$) interactive methods performed significantly (all $p <=.010$) better than *Video* training ($M = 58.57$, $SD = 22.89$).

PD: “*Physical demand - How physically demanding was the task?*” The Welch ANOVA found a significant difference in perceived physical demand $F(4, 64.793) = 5.008$, $p < .001$, $\eta ^{2} = 0.093$. Participants’ physically demanding feedback was significantly ($p =.026$) lower in the *MA* training ($M = 10.18$, $SD = 7.99$) than in the *VR* training ($M = 23.39$, $SD = 16.84$).

TD: “*Temporal demand - How hurried or rushed was the pace of the task?*” The Welch ANOVA indicated significant differences between the conditions regarding the temporal demand $$F(4, 66.684) = 15.141,$$ $$p <.001$$, $$\eta ^{2} = 0.355$$. Pairwise comparisons showed that all methods were significantly (all $$p <=.005)$$ lower compared to the *Video* training ($$M = 63.21$$, $$SD = 27.36$$). Also, the temporal demand for the *MA* ($$M = 23.21,$$ $$SD = 15.53$$), *VR* ($$M = 20.89$$, $$SD = 18.51$$), and *AR* training ($$M = 22.68$$, $$SD = 23.39$$) was significantly (all $$p <=.018$$) less compared to the *Group* training ($$M = 41.96$$, $$SD = 25.22$$).

P: “*Performance - How successful were you in accomplishing what you were asked to do?*” The statistical comparison of the participant’s perceived performance differed $F(4, 135) = 5.887$, $p <.001$, $\eta ^{2} = 0.149.$ Participants of the *Group* ($M = 48.39$, $SD = 19.72$) and *Video* training ($M = 48.21$, $SD = 18.92$) rated their performance significantly (all $p <= .048$) lower compared to the *MA* ($M = 31.79$, $SD = 25.79$), *VR* ($M = 26.96$, $SD = 20.83$), and *AR* training ($M = 31.61$, $SD = 24.54$).

E: “*Effort - How hard did you have to work to accomplish your level of performance?*” In terms of effort, the Welch ANOVA showed a difference $$F(4, 66.925) = 7.485$$, $$p <.001$$, $$\eta ^{2} = 0.192$$. Participants in the *Video* training ($$M = 44.64$$, $$SD = 23.25$$) had the highest effort, which significantly (all $$p <=.010$$) differed compared to the *MA* ($$M = 20.89$$, $$SD = 14.97$$), *VR* ($$M = 20.36$$, $$SD = 15.81$$), and *AR* training $$(M = 27.14$$, $$SD = 19.69).$$ The pairwise comparison also showed an effect (all $$p <=.023$$) for *Group* training $$(M = 36.96$$, $$SD = 23.31),$$ where the participants’ effort was higher than *MA* and *VR* training.

F: “*Frustration - How insecure, discouraged, irritated, stressed, and annoyed were you?*” Statistical comparison using Welch ANOVA showed that frustration differed between groups $F(4, 66.408) = 8.225$, $p <.001$, $\eta ^{2} = 0.161$. It was highest rated in the *Video* group ($M = 43.57$, $SD = 28.31$) and showed a significant (all $p <= .039$) difference compared to the *MA* ($M = 17.32$, $SD = 15.24$), *VR* ($M = 25.54$, $SD = 27.63$), and *AR* ($M = 24.64$, $SD = 23.45$) groups. There was also an effect ($p <= .002$) between the *Group* ($M = 41.25,$ $SD = 21.15$) and *MA* training.

O: “*Overall*” We further analyzed the overall workload resulting from the mean of the six subscales with a one-way ANOVA that showed a clear difference between the five training methods $$F(4, 135) = 17.095,$$ $$p <.001$$, $$\eta ^{2} = 0.336$$. The workload was highest for the *Video* training ($$M = 46.01$$, $$SD = 16.14$$) and differed significantly (all $$p <.001$$) from the *MA* ($$M = 22.77$$, $$SD = 10.03$$), *VR* $$(M = 24.61$$, $$SD = 11.51),$$ and *AR* training ($$M = 27.80$$, $$SD = 13.06$$). There was also an effect (all $$p <=.029$$) between the *Group* training ($$M = 37.77$$, $$SD = 11.31$$) compared to the *MA*, *VR*, and *AR* training.

### Online test

**Fig. 5:**
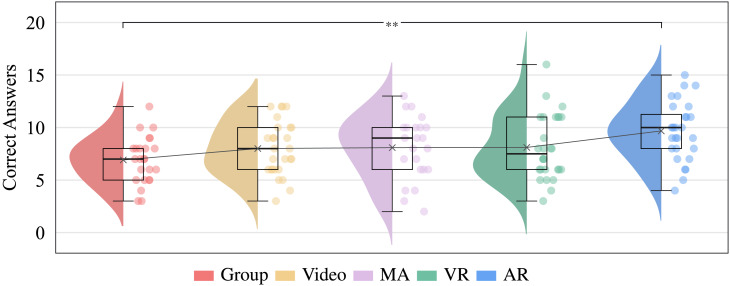
Mean values of the correct answers on the knowledge test. The connected crosses represent the mean scores.

Of the initial 140 participants, 130 completed the online test ($$92.86\%$$ response rate) to measure training success. An ANOVA was used for the statistical analysis since, despite the unequal sample sizes, the assumptions of normal distribution and homogeneity of variance were fulfilled. The comparison of average correct answers between groups showed a significant difference $$F(4, 125) = 3.372$$, $$p = .012$$, $$\eta ^{2} = 0.097$$. The Tukey-Kramer post-hoc test indicated that the participants of the *AR* training ($$M = 9.68$$, $$SD = 2.79$$, $$n = 28$$) answered significantly ($$p =.004$$) more questions correctly compared to the *Group* training ($$M = 6.92$$, $$SD = 2.24$$, $$n = 24$$). Figure 5 illustrates that participants in *AR* training also performed better than *VR* $$(M = 8.11$$, $$SD = 3.07$$, $$n = 28),$$ *MA* ($$M = 8.09$$, $$SD = 2.98$$, $$n = 23$$), and *Video* training ($$M = 8.00$$, $$SD = 2.57$$, $$n = 27$$) participants.

## Discussion

### Summary of results

 The results clearly show that training can be completed in the shortest amount of time using the *MA* or *Video* method. In contrast, *VR* and *AR* training require the most time. Despite the same training content, the conventional *Group* training structure (Q1) is significantly the lowest. The more engaging topic preparation (Q2) in the *MA*, *VR*, and *AR* methods leads to increased motivation. Among all methods, *AR* training is the most realistic (Q3) and differs significantly from *MA* training. The conditions content quality reveals no statistical effect (Q5), but considering the overall workload (NASA-RTLX), there is clear evidence that *MA*, *VR*, and *AR* training outperform the other two methods. In the training conditions’ overall rating (Q6), the *VR* and *AR* methods dominate and perform significantly better than *Group* and *Video* training. *VR* and *AR* training also receive the best subjective training success (Q4) ratings. Inter alia, *AR* training is significantly better than *Group* training. This is consistent with the online test results, in which the *AR* training also performs significantly better compared as those in *Group* training. Of the remaining conditions - *Video*, *MA*, and *VR* training - the online test’s error rates are roughly the same.

### Explanation of findings

 We attribute that the *AR* condition’s training duration is the longest since the effort required for priming the physical machine is more extensive (*e.g*., inserting the hoses is more complex) than the virtual execution or its observation. The short training time required for the *MA* and *Video* conditions can be attributed to the participants’ motivation since, in the *Video* condition, participants see no need to further engage with the topic or re-watch the video. *Video* training only offers the possibility of interaction by controlling the video, which negatively affects intrinsic motivation compared to interactive training systems^49,50^. The time difference between the *Group* and the *Video* training can be explained by the fact that the expert also answers questions during conventional training. In the *MA* condition, participants’ interest decreases after a few steps as they focus more on quickly completing the steps and less on the content. We attribute this phenomenon to the participant’s familiarity with the established technology and the uncomplicated interaction (tap, drag-and-drop), which is not challenging to the participants and thus reduces their attention^51^.

In all training methods, the same training content is presented to the participants in the same order. However, the training structure (Q1) is significantly the worst in the *Group* condition. This is attributable to users’ ability to follow the instructions better in the other conditions, as the training speed is controlled by completing a step (*MA*, *VR*, or *AR*) or using the functions of a video player (*Video*)^52^. In addition, *Group* and *Video* training are perceived as less interesting (Q2) than the other methods, negatively affecting participants’ motivation^53^. As anticipated, practical feasibility (Q3) is rated best in *AR* training, although a significant difference exists compared to *MA* training. We assume this is due to the higher presence level of the *AR* training compared to the other methods, as the dialysis machine is perceived more realistically^54^.

Despite the identical training content and the fact that the methods are implemented comparably as far as possible, the content quality (Q5) is perceived differently, *e.g*., since participants of the *MA*, *VR*, or *AR* training have the opportunity to examine certain details from different perspectives, whereas this is only possible from one perspective during *Group* and *Video* training. However, the statistical evidence is not sufficiently strong to conclude that the content quality differs between the groups.

The overall workload of the RTLX is lowest for the *MA*, *VR*, and *AR* conditions since five of the six RTLX subscales are rated highest for *Group* and *Video* training. Only the physical demand subscale ratings are roughly equal to those of the other methods. One explanation is that observing an instructor or watching a video requires less physical activity than interacting with a virtual or physical device. The RTLX subscale temporal demand rating of the *Video* group could indicate that the playback speed of the video is too fast for the participants. However, we assume the participants are not overwhelmed as they have no time limit and can perform functions, *e.g*., pausing or rewinding the video.

For the *VR* and *AR* training, participants rate their ability to prime the dialysis machine without any problems (Q4) significantly better than for the other methods. This is consistent with the overall training (Q6) ratings and the results of other contributions^28,33–35^. Overall, the results of the subjective training success can be attributed to the fact that training with *VR* and *AR* is easier and more motivating for the participants^55^. Moreover, this is supported by the online test results, according to which the *AR* training shows a significantly better training outcome than the *Group* training. Our results from the *VR* group agree with those of Winther *et al*.^31^ regarding the longer training duration but not the higher error rate. Furthermore, the results of the online test, where only $43.25\%$ are correct, support the statement from other studies that *Group* training is insufficient^1–9^. Regarding the results of the other methods, it should be noted that *AR* training, *e.g*., not only performs $17.25\%$ better but also has the potential to achieve better results in the future, as the technological possibilities of this and the other digital methods have not been exhausted due to the prototypical and comparable development.

The findings of our investigation serve to answer the following research questions:

RQ 1: Can alternative training methods improve medical device training?

The results demonstrate that alternative methods perform better in workload and both subjective and objective training success compared to conventional *Group* training. Additionally, the participants’ positive ratings (Q1, Q2, and Q6), combined with the known demand^1^ and interest^19,20^ in new medical training approaches, emphasize the potential of these technologies and user acceptance^56^, which is also a decisive factor.

Aside from this, these technologies can also perform complementary roles to conventional training. For instance, allowing users to interact with the device independently and without the pressure of time or success after training. This possibility not only promotes familiarity with the device but also ensures compliance with statutory regulations (*e.g*., FDA (https://www.fda.gov), MDR (https://health.ec.europa.eu), or TGA (https://www.tga.gov.au)) for medical device usage. Consequently, alternative training methods hold considerable potential to improve medical device training in various respects.

RQ 2: Do alternative medical device training methods differ in terms of training success?

The results of the *VR* and *AR* methods are significantly better than those of the other methods. However, *AR* training is the most suitable since it provides the best objective training success in the online test. Users completing *AR* training on a physical device are more familiar with it than with training on a virtual simulation. Especially since the *Video* and *MA* training performs worse regarding the study results, its application possibilities also offer no severe advantages compared to the *VR* and *AR* training.

### Implications for design

 Our findings demonstrate that alternative training methods have the potential to be used for medical device training. Each method has specific advantages and disadvantages that need to be considered for future application. One aspect is that a physical dialysis machine and its equipment material are only required *Group* and *AR* training. Therefore, unlike the other methods, these can only be performed in a medical facility (unless it is a home dialysis machine^57^), and only if the required dialysis machine is available. In exceptional situations, such as pandemics, many patients may suddenly require treatment on rarely used medical devices. This creates bottlenecks in the availability of these devices and their associated materials, leading to a conflict between the patient’s treatment and the medical staff’s device training^58^. The remaining three methods can be completed in any location. The advantages of all training methods over *Group* training are that users can complete the training at any time, without time pressure, without distraction from others, as often as they want, in multiple languages, and in a standardized way.

Another aspect is the level of interaction and exploration during the study’s priming procedure. The *Group* and *Video* training are limited compared to the other methods, as the users can only observe the interactions performed by an expert and are not allowed to interact or explore (*e.g*., from different perspectives) the real or virtual device. In the *MA* training, the priming is simulated via a 2-dimensional interaction with a touch display. While in *VR* training, the interaction is more realistic since the users can grab, move, and place virtual objects in a 3-dimensional space. However, specific details, such as the haptic feedback when placing or clamping objects, are missing. The *AR* training interaction is the most realistic as it corresponds to a real priming situation. Users receive instructions audibly and visually superimposed on the physical device, allowing them to perform all training steps simultaneously on the device.

Given these findings, it is evident that of all the methods investigated, *VR* and *AR* have the highest potential to improve medical device training by complementing or even replacing conventional training. In the best-case scenario, this could save human and financial resources^3^ and increase patient safety. Therefore, a more specific investigation is required to evaluate the effectiveness of these methods, including assessing the participants’ ability to prime the real device without assistance. Considering the limited development resources for these research applications, we are very optimistic and believe that integrating more details, such as an avatar guide^59,60^, different training levels^61,62^, or gamification techniques^63,64^, could improve the applications not only didactically but also in terms of user experience.

However, it should be noted that the *Video* and *MA* methods also offer specific advantages. One major advantage of *Video* training is the ability to produce and distribute the relevant training content with the least effort. With additional effort, the training outcome could be improved. One way to achieve this is by adding interactive functions to the *Video* training, such as allowing users to select placeable objects or their target positions for controlling the video sequence^65^. Similarly, *MA* training could be improved with video content so that users interactively perform a training step on the mobile application (*e.g*., tablet or smartphone), followed by a short video clip demonstrating a person performing the corresponding step on a physical dialysis machine.

### Limitation and future work

 Our study focuses on providing empirical evidence on the effectiveness (*i.e*., training success) of alternative training methods for medical devices. For this purpose, we investigate four alternatives to the conventional training method. These are specified based on the feasibility criteria, but alternative options exist, such as In-situ^36^ or a Cave Automatic Virtual Environment^43^, which are not investigated. The training success is assessed by subjective evaluations and an online test, so the training success could vary when evaluating on a real machine. As a test is a common and established scientific method for assessing training success, only minor deviations are to be expected. These are tolerable, as the focus of this work is to identify whether and which alternative training methods have the potential to increase training success in order to investigate them specifically and comprehensively in future work. In addition, evaluating the training success of all five methods on a real device would have been much more problematic due to logistical requirements such as available patient rooms with special connections and new equipment materials for each participant.

Another limitation mentioned in section 4.5 is that the *AR* training is performed on a real disabled dialysis machine, which does not seriously impact the results. The *Video* and *MA* training also offers the opportunity to be performed parallel to the physical priming procedure of the dialysis machine. This approach is similar to *AR* training but with some differences. In *Video* training, users first watch a training step and then pause the video to perform the same step on the physical machine. Meanwhile, in *MA* training, users interactively perform the respective training step on the mobile application, followed by the physical device. As this could affect training success, we recommend investigating this in future work. To this end, the concepts of both methods should be revised, for instance, by positioning the mobile device (*e.g*., tablet or notebook) next to the physical dialysis machine. So, users are not required to hold the mobile device in their hands and can keep their hands free, as in *AR* training. In addition, the short distance between the dialysis machine and the mobile device avoids unnecessary walking back and forth.

Future work could also investigate more complex interactions between virtual and real objects, also known as mixed reality (MR)^66^. Mixed reality can be interpreted in various ways, such as AR or VR^67^, as well as other ways^68^. Thus, the technological possibilities are outlined below. Networking between the medical device and the training device (*e.g*., Apple Vision Pro (https://www.apple.com/apple-vision-pro), Meta Quest 3 (https://www.meta.com/quest/quest-3), or Microsoft HoloLens 2 (https://www.microsoft.com/hololens)) enables the transmission and processing of information. Since medical devices such as the dialysis machine used in our experiment can recognize correctly inserted objects. If this information is transmitted to the AR/MR HMD, the following training step can be executed automatically instead of performing voice commands. In addition, AR/MR HMDs can capture environmental information, *i.e*., the required components for priming can be recognized by object detection, resulting in alternative visualization options such as highlighting the physical objects or routing to their destination position.

Our investigated training methods also indicate exciting opportunities for patient training. In particular, specific therapies, such as hemodialysis, can be carried out by patients or caregivers at home. This approach offers several advantages. In the long term, it is a more cost-effective treatment option^69,70^, it improves patients’ quality of life by eliminating the need for frequent and time-consuming visits to medical facilities^57,71^, and it has the potential to improve treatment outcomes by allowing patients to dialyze more frequently and for longer periods of time on a flexible schedule^71^. Despite the advantages of home dialysis, utilization rates remain low due to patient concerns and fears about self-administration^72^. To address these issues, it is necessary to investigate the potential of alternative or complementary methods for patient training. The AR/MR capabilities mentioned above can be used to monitor the patient’s actions to prevent potential errors by directly communicating them to the user during the device priming procedure.

The costs for the hardware (*e.g*., between a tablet, VR, or AR headset) and for developing the individual training method (*e.g*., producing a training video compared to implementing an AR application) vary considerably. As the development costs are incurred only once per medical device and the training can then be used worldwide, it is quite conceivable that (depending on market demand) alternative methods are more economical, as each conventional training course conducted by a manufacturer’s expert costs several hundred to thousand dollars.

## Conclusion

 Medical device training has been a persistent issue for many years, negatively impacting patient safety. In order to address this challenge, this contribution presents four alternative training methods for the priming of a dialysis machine and investigates their training success using a between-subjects study ($n=140$). This involved comparing the training methods *Group*, *Video*, *MA*, *VR*, and *AR*. The *Group* training was equivalent to conventional training. The results show that both the subjective and objective training success are significantly better in the *AR* group than in the conventional *Group* training. In contrast, the *Group* training currently used in medical facilities achieved the lowest training success in an online test. All findings indicate that alternative training methods, particularly *VR* and *AR*, have the potential to improve medical device training and improve patient safety.

Based on our research findings and the long-standing expert recommendations for integrating better training methods, we strongly recommend supplementing conventional training with *AR* or *VR* methods. This supplementation initially requires no changes to the legal requirements, allows medical professionals to familiarize themselves with digital training, and offers the opportunity to clarify any ambiguities and uncertainties that still exist after the completion of conventional training. This would be an essential first step to address the challenges and examine these methods’ effectiveness in real-life scenarios.

## Method: dialysis training

In our work, we focus on the priming process of a hemodialysis machine. This device is used in cases of kidney failure to act as an artificial kidney that cleans the patient’s blood. We use the 5008s Cordiax dialysis machine manufactured by Fresenius Medical Care (https://www.freseniusmedicalcare.com/en/5008s-cordiax), which is used daily by the medical staff and accessible to us. To ensure that the device training is comparable between all conditions, the manufacturer’s manual and a medical nephrology expert serve as input to define the training content. In total, this results in 60 relevant training steps integrated into all five training methods. Out of these, seventeen steps are informative, meaning they convey specific details to the user, such as verifying a connection or conveying theoretical details of a task. The remaining 43 steps require direct user interaction with the dialysis machine. An excerpt of these steps can be taken from Table 3.

In the training methods, *Group* and *Video*, no interactions are allowed, so users can only observe the execution of these steps. In contrast, interactions of *MA* and *VR* conditions are carried out virtually and in *AR* training on a physical dialysis machine. For these methods, we take care that users are not distracted by complex interactions^73^ or graphical details. Furthermore, tutorials are also implemented for *MA*, *VR*, and *AR* training to ensure users can handle the technology and interaction techniques.

During the *Group* training, the users can hear the defined training steps by the instructor; for reasons of comparability, we integrated recordings of a professional speaker into the other four training methods. In the *MA*, *VR*, and *AR* conditions, the content of the voice recordings is also displayed visually as text for effective information transfer^74^. This allows users to re-read the instructions if they were, *e.g*., distracted by the interaction. In contrast, the *Video* condition provides no text/subtitles to achieve a better training effect^75^. Additional three-dimensional (3D) models of objects, such as the dialysis machine and its components, are reconstructed to create an immersive 3D environment. For this, we use the 3D creation suite Blender (https://www.blender.org). With these 3D models, the voice recordings, and the defined steps, we implement the *MA*, *VR*, and *AR* applications using the Unity development environment (https://unity.com). An overview of the differences between the conditions is depicted in Table 2 and explained in more detail below.

**Table 2:** Overview of the differences between the conditions.

Method	Instructions		Interactive objects		Visual objects	
	Auditive	Visual	Device	Components	Device	Components
Group	Live	-	-	-	Physical	Physical
Video	Recording	-	-	-	Physical	Physical
MA	Recording	Text	Virtual	Virtual	Virtual	Virtual
VR	Recording	Text	Virtual	Virtual	Virtual	Virtual
AR	Recording	Text	Mixed	Physical	Physical	Mixed

Instructions are conveyed audibly and/or visually to the user. Interactive objects indicate if the user can interact with the dialysis machine or its components, and visual objects indicate how the user perceives them. Mixed is a combination of physical and virtual.

**Table 3:** An extract of the steps performed by the participants for priming the dialysis machine.

ID	Interaction task	ID	Interaction task
1	Insert dialyzer	2	Insert citrasate canister
3	Connect canister with hose	4	Insert sporotal
5	Connect sporotal with tubing	6	Activate switch behind the machine
$\vdots$	$\vdots$	$\vdots$	$\vdots$
34	First, place the other end at the bottom of the device	35	Pull and turn the irrigation port counterclockwise
36	Attach the irrigation plug to the irrigation port	37	Connect the irrigation port
38	Turn and pull substitute port to attach substitute plug	39	Connect one end of the SafeLine to the substitute port
40	Close the substitute port	41	Attach red drain hose to lower part of the filter
42	Attach blue inlet hose to the upper part of the filter	43	Close the device doors

These steps are identical in all five training methods, but in the Group and Video training, they are not performed interactively by the participants but demonstrated to them.

### Group training

This condition serves as a baseline and corresponds to traditional medical device training, *i.e*., it occurs under the same conditions (*e.g*., the training content and duration). Here, a group of medical professionals is trained simultaneously by a nephrology expert, who explains all defined steps and performs them live on the dialysis machine. This typical procedure resembles the one depicted in Fig. 1. Just like participants of a traditional training, study participants are allowed to ask the instructor questions during the training. Due to pandemic hygiene regulations, the training occurs in a lecture hall. As the spatial conditions and the seating arrangement offer the advantage that the participants have a clear view of the training content, this has no negative impact on the evaluation. The distance to the dialysis machine is approximately 2 to 5 meters, enabling participants to observe all details.

### Video training

**Fig. 6:**
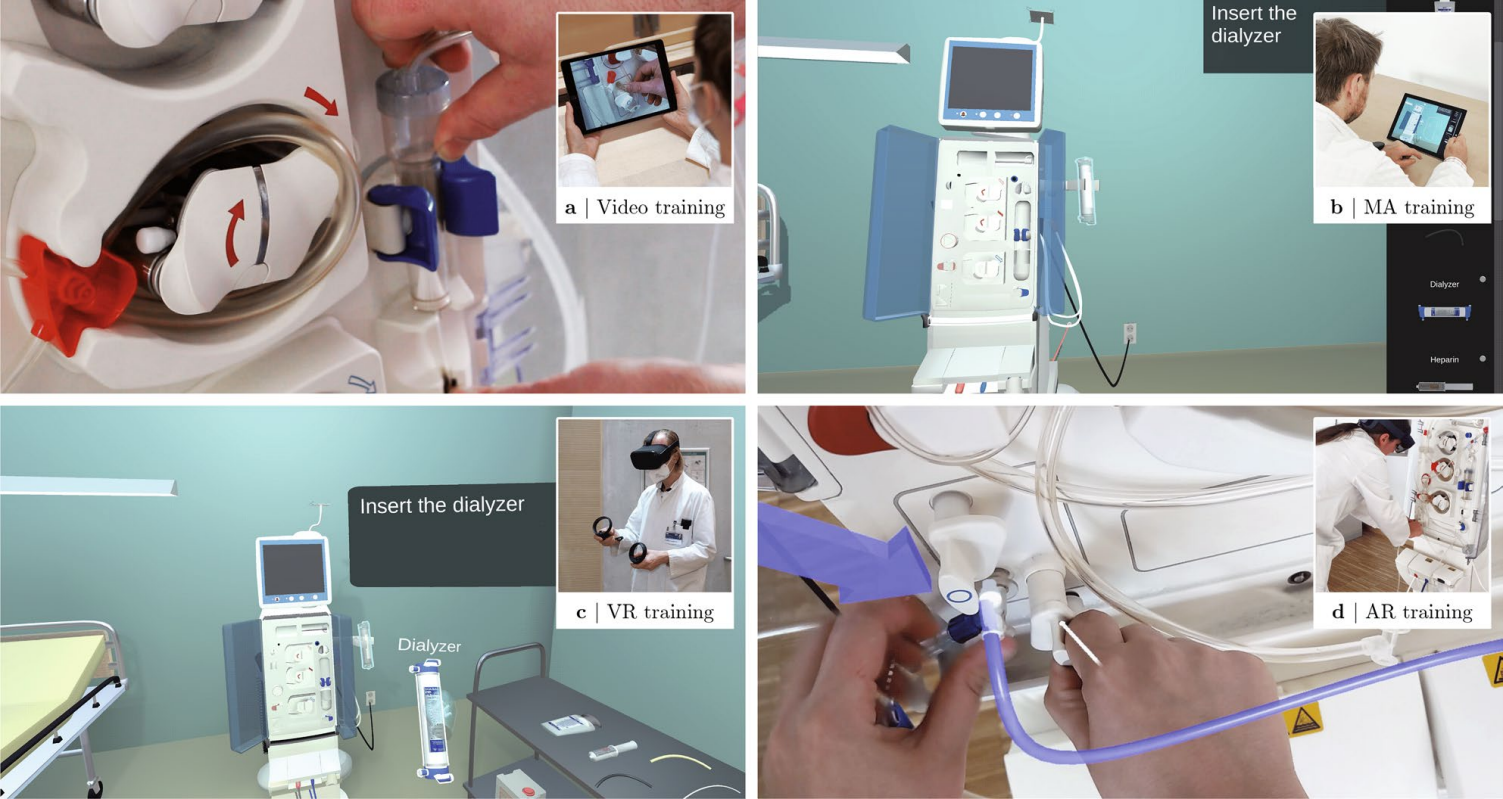
Excerpt from the alternative methods: (**a**) Video training - the user watches a video, (**b**) MA training - the user drags the virtual objects to their correct position, (**c**) VR training - the user is in a virtual environment where he primes a virtual dialysis machine, and (**d**) AR training - the user executes the visually augmented tasks on a real dialysis machine.

In this method, trainees watch a video that explains the defined steps. For the visual content, we recorded a dialysis expert priming the machine. We edit this content and supplement it with recorded instructions and explanations of the defined training steps voiced by a professional speaker. Participants watch this video on an iPad, as shown in Fig. 6. They are allowed to fast-forward and rewind the video, watch it several times, and adjust the volume.

### MA training

For this approach, participants train with a mobile application operating on a Microsoft Surface Pro, as illustrated in Fig. 6. Interaction occurs step by step by virtually placing the components at the correct device position. Placement is performed by putting the finger on the component, dragging it to the correct device position, and lifting the finger to drop it. When the component is close to the correct position, it snaps into place and aligns itself to the intended position, then the next step occurs. If it is misplaced, it reappears at its original position. Some training steps require the user to tap a specific virtual position on the screen, such as a power switch on the virtual device. Steps that do not require direct interaction with the virtual machine display a continue button on the screen. All components in the list have an icon that, when clicked, provides a detailed view with zoom and scroll functions.

### VR training

This method uses the Oculus Quest (https://www.oculus.com/quest) all-in-one VR glasses. Software is implemented with the same 3D models, voice recordings, and training steps as in the other applications. The interaction principle is similar to the *MA* application, except that two 6-degree-of-freedom controllers are used instead of a touch screen. As depicted in Fig. 6, virtual components can be transformed by pressing, holding, and releasing one of the controller’s trigger buttons. Collisions between the controller position and a virtual object activate virtual switches. Informative steps that do not require direct interaction are completed by pressing the x-button on the left controller. Since the dialysis machine is usually used in a patient’s room, the virtual environment of the *MA* and *VR* training mirrors this to increase the user’s immersion and mental performance^76^.

### AR training

The Microsoft HoloLens 2 HMD (https://www.microsoft.com/hololens) is used for the last training method. This allows users to train with the real dialysis machine by seeing the relevant training information augmented at the physically correct position, as visualized in Fig. 6. So, users train with the real equipment and place it step by step at the corresponding positions according to the 3D visualization. They also press the switches on the device, whose positions are displayed in the AR glasses. The software does not recognize when a task has been completed, so the next step must be manually called by pressing a virtual button next to the dialysis machine. Tasks are also communicated audibly and visually. As in the *MA* and *VR* application, the visual cues are located at the top right, next to the dialysis machine. The materials to be placed are on a table to the right of the dialysis machine, as in the *VR* training. Only one priming set is used for the study to avoid unnecessary material waste. For this reason, the training device is not connected to the power supply to prevent, *e.g*., the automatic filling of the materials with liquids, which usually occurs during the priming procedure. As a result, the dialysis machine’s monitor is not working, so we use the AR glasses to simulate it at the correct position.

### Study design

**Table 4:** Questions that participants assessed after completing one of the five training conditions.

ID	Subjective evaluation question	Category
Q1	I think this training was clearly structured.	Structure
Q2	I think the training topic was interestingly prepared.	Motivation
Q3	I think the training was close to reality.	Practical feasibility
Q4	I feel able to prime the dialysis machine without any problems.	Training success
Q5	Which grade would you give the quality of the content?	Content quality
Q6	Which grade would you give the training overall?	Overall assessment

Q1 to Q4 were rated using a 7-point Likert scale [1 = strongly disagree, 7 = strongly agree], and Q5 to Q6 using a 6-point Likert scale [1 = very good, 6 = not sufficient].

We conducted a between-subject study design since participants were tested regarding their training outcomes, meaning they could only participate in one of the five conditions. These conditions were the *Group*, *Video*, *MA*, *VR*, and *AR* training, which constituted the independent variable. As dependent variables, we collected participants’ training completion time and ratings on specific questions presented in Table 4. Questions Q1 to Q3 are taken from the Feedback Instrument for Rescue Forces Education Questionnaire (FIRE-B)^77^. Furthermore, we asked participants to complete the Raw NASA Task Load Index Questionnaire (RTLX)^78^ to assess their perceived workload. One week after completing the training, participants received an online test via e-mail to measure the training’s success. Medical device training participants usually do not handle the device immediately after the training but often at irregular intervals, depending on the device. Therefore, the online test took place after one week to assess the crucial long-term knowledge^79,80^. The test consisted of 16 questions, which are listed in Table 5. These were single-choice questions with three possible answers and matching questions in which five items/labels were assigned to five markers in a figure. Each assignment was defined as one question, resulting in $2\times 5$ questions for a comprehensible evaluation of the matching question results.

**Table 5:** Questions of the online test for assessing the training success.

ID	Online test question	Type
1	How many switches/buttons are operated to turn on the device?	Single-choice
2	Which hose system is connected to the SafeLine?	Single-choice
3	Which blood pump is used for priming?	Single-choice
4	Which object is attached to the device first?	Single-choice
5	Where are the two tubes attached to the dialysis machine’s side connected?	Single-choice
6	What is the step after which the priming of the device is complete?	Single-choice
7-11	Assign the correct items to the markers in the image.	Matching question
12-16	Assign the correct technical terms to the marker in the image.	Matching question

## Data Availability

All data collected during this study are available upon request from the corresponding author (Maximilian Rettinger).
